# Contextualizing chronicity: a perspective from Malaysia

**DOI:** 10.1186/1744-8603-8-4

**Published:** 2012-03-08

**Authors:** Shajahan Yasin, Carina KY Chan, Daniel D Reidpath, Pascale Allotey

**Affiliations:** 1 Global Public Health, Jeffrey Cheah School of Medicine and Health Sciences, Monash University Sunway Campus, Kuala Lumpur, Malaysia

## Abstract

The increasing prevalence of chronic Non Communicable Disease (NCD) around the world is well documented and projections suggest a frightening increase in prevalence around the world. The majority of new patients with chronic disease are expected to occur in developing countries.

Effective management of chronic disease is a complex process that involves a proactive health care team working within an integrated healthcare delivery system supporting a well informed and confident patient skilled in self-management of the condition.

There is increasing evidence especially from western countries that methods of implementation that use these principles work.

Widespread and not contextualized dissemination of these approaches especially to less developed countries, however, would pose particular challenges. These challenges relate to a number of factors; a lack of resources, poorly functioning healthcare systems and their ability to cope, the rise of private financing for healthcare with increasing out-of-pocket payments for accessing healthcare, rapid industrialization and urbanization with attendant breakdown in support relationships and the general lack of support services including a social support model.

We discuss some of these health system issues, using diabetes as the indicator condition, and the relating this to the Malaysian health system to illustrate the challenges of translating evidence from better resourced countries. Malaysia is a middle-income country with a well-functioning public health system designed primarily for control of communicable disease and Maternal and Child health. While a population approach in dealing with NCDs is key, we have highlighted an individual high-risk approach in this commentary.

A number of patient support systems by professionals have been tested successfully in developed countries. In most developing countries, individuals especially the elderly depend on families to provide support. This and support from peers may be areas that may require further study especially in the area of self-management.

## Introduction

Chronic Non Communicable Diseases (NCDs) are the leading cause of mortality in the world accounting for over 60% of all deaths - 35 million deaths each year with 80% of these occurring in developing countries [1]. The WHO predicts that global mortality from chronic diseases will rise by 17.6% between 2006 and 2015. This increase will be distributed unevenly with an increase of 4% in Europe and 17% in America whereas in the low-and-middle-income regions the increase is estimated to be as high as 27% in Africa, 20% in the Western Pacific and 21% in South East Asia [2].

The prevalence of specific chronic diseases such as diabetes, hypertension, stroke and other cardio vascular conditions is of even greater concern [3]. There are approximately 250 million people worldwide living with diabetes; by 2025 this figure is expected to grow to 400 million; with 75% of these in developing countries [4]. There were 12.9 million cases of cancer globally in 2009 with numbers expected to double within a decade. Approximately 60% of these new cases are projected to come from low-and-middle-income countries [5].

The rise of NCDs in South East Asia has been recently highlighted [6]. Up to 60% of deaths in the region can be attributed to NCDs [7]. It is estimated from the Malaysian Third National Health and Morbidity Survey in 2006 that 70% of Malaysian adults suffer from a NCD like diabetes, hypertension and cancer and NCDs account for 51% of deaths in the country [8]. Risk factor prevalence in Malaysia, especially physical inactivity, at 15%, is the highest among South East Asian countries [9].

Malaysia is among the top ten countries in the world with high percentage of adult population living with diabetes at 11.6%. Among adults over 30 years of age, overall prevalence of diabetes has nearly doubled to 14.9% from 8.2% in 1996. The prevalence of the disease increases to 50% among persons aged 60 and above [9].

The prevalence of risk factors also highlights the significant nature of the problem in Malaysia and other low-and-middle-income countries. In broad terms it is understood that these trends in chronic diseases can, in part, be attributed to industrialization, urbanization and replacement of a more rural, active and traditional lifestyle with a more sedentary lifestyle, often involving less activity and a less healthy diet. Most approaches to the prevention and management of chronic diseases therefore call for changes in lifestyle; better diets, increase in physical activity, avoidance of exposure to environmental carcinogens and reducing high risk behaviours such as smoking.

Health service approaches to managing NCDs explore challenges of access to screening for risk factors, regular monitoring and provision of expert care mostly centred on acute care facilities [5]. Ultimately, these approaches are based on an understanding of interventions at the level of the individual. However, given the nature of the 'epidemic', the chronicity of chronic diseases requires a redefinition of what we currently take for granted as 'normal' or 'healthy'. With whole populations at risk conceptualizing and addressing chronic diseases cannot focus on current paradigms of illness and health seeking. When faced with such numbers it is critical that we think beyond individual care to the implications for societies of having large numbers of people with chronic health conditions.

A number of approaches to this problem have been proposed and are being implemented in various settings. There is also evidence on the effectiveness of these approaches. It is important to note, however, that the chronicity of chronic diseases means that prevention and management is heavily context-dependent. With a focus on Malaysia, we explore the implications of chronicity as they relate to the distribution of chronic disease across the population, and the challenges posed to current approaches to prevention, treatment and management. While control of high prevalence NCDs diseases would involve approaches at a population level as well as at individual level, we would be concentrating on the individual approaches with particular emphasis on health system issues. We have used Type 2 diabetes mellitus for illustrative purposes as the indicator condition due to the high prevalence in Malaysia as well as the complexity of management of this condition.

### The Malaysian Health System

The Malaysian health system is a publicly funded system which has been successful at a moderate cost in dealing with the major public health priorities of developing countries including communicable disease as well as maternal and child health issues. The public health system caters for the health care needs of the majority of the population.

However, the private sector plays a significant role in healthcare especially in the urban areas. This system is privately financed and, especially in urban areas, replicates the public system from primary care general practitioner clinics to tertiary care centres. It has seen significant growth in recent years. The private sector has a stronger focus on providing curative services as opposed to public health services. In 2002, 44% of health care expenditures were raised privately, mainly through out-of-pocket payments. Private practitioners service about 57.1% of outpatient visits and private hospitals see 17.9% of inpatient encounters [10]. (See Table 1: Malaysian Health System)

**Table 1:** Data showing Malaysian Demographics, Economic, Health Status and Provision of Health Services

Indicator (unit)	2006*
Gross Domestic Product (Ringgit Million, Constant Prices)	277,263

Population (millions)	27

Crude Birth Rate (number of births per 1,000 population)	18

Crude Death Rate (number of deaths per 1,000 population)	4.5

Life Expectancy at Birth (years)	71.6 (male), 76.2 (female)

Infant Mortality Rate (number of infant deaths per 1,000 live births)	6.7

Maternity Mortality Rate (number of maternal deaths per 100,000 live births)	30

Hospital Beds per 1,000 Population	1.45 (public), 0.44 (private)

Physicians per 1,000 Population	0.50 (public), 0.32 (private)

Nurses per 1,000 Population	1.90 (public), 0.51 (private)

N.B. *Data extracted from "Health financing note East Asia and Pacific region" [11]

### The Malaysian Public Health System

The public health system in Malaysia is built on the WHO model for district health systems. It is made up of semi-autonomous health districts (or sub-districts) which provide comprehensive primary health care to defined catchment population within clear geographical boundaries.

The public health care facilities within a typical district would consist of a district hospital and a number of large primary care clinics or Klinic Kesihatan (KK) run mainly by medical generalists (family medicine specialists) and nursing personnel. It provides ambulatory primary care for the catchment population including acute medical and surgical presentations for adults and children, antenatal and postnatal care and management of chronic non-communicable diseases. Most KKs are equipped with X-rays and ultrasound as well as basic laboratories capable of doing urine and blood examinations.

Linked to, and one step down from the KKs in each subdistrict, are three or four smaller community clinics or Klinik Desa (KD), run exclusively by nurses and midwives. Each KD is responsible for all households within sub-populations of 4,000 to 5,000 people for whom they provide preventive Maternal and Child Health (MCH) services, including postnatal follow-up and care for mothers and newborn infants, family planning, cervical screening, immunisation and child health monitoring for preschool children. Home visits, especially for antenatal and postnatal care, form an integral part of the work of community nurses.

This system has explicit public health priorities, such as communicable diseases and maternal and child health. These have been effectively targeted with dramatic reductions in infant and child mortality and there has been a steady improvement in life expectancy over the past thirty years [12].

We now discuss some effective strategies for NCD management before returning to some ways in which the Malaysian health system have targeted these conditions.

### Systems approach to NCD management

A number of approaches have been proposed for the management of chronic disease with a specific focus on working through communities. The systems approach involves a redesign of the healthcare delivery systems to make it responsive to the chronic or long term nature of these conditions. The chronic care model developed by Wagner and described by Bodenheimer et al. (2002) in their systemic review, involves improvements in six areas: the community, the health system, delivery system design, decision support, clinical information systems and support for self-management [13,14]. The model is based on the notion that informed and activated patients interacting with prepared, proactive practice teams leads to productive interactions and better outcomes. Patients become informed and activated through effective support of self-management [15]. The Innovative Care for Chronic Conditions (ICCC) Framework, adapted from the Chronic Care Model (CCM) [16] in 2002, identifies core building blocks to redesign health care systems to cope with long-term health conditions. ICCC was developed, recognizing the challenges of the under-resourced and non-integrated health systems in low-and-middle-income countries. The framework relies on partnerships that support patient and family interactions at the micro level; health care organization and community; and a well-coordinated policy and health systems environment.

### Role of healthcare systems

Strengthening of health systems especially to deal with NCDs has become an increasing focus in recent times [17-19]. Health systems in middle-income countries face financial, resource and personnel constraints. These countries are going through an epidemiological transition and are fighting on two fronts: while chronic diseases are important causes of mortality, infectious diseases remain significant part of the disease burden.

Chronic illness management requires health systems to be able to deliver integrated care across multiple disciplines with collaboration across sectors. Such collaboration is required between the public and private health sectors as well as between curative services and the public health or preventive services.

### Implementing an ICCC Model in Malaysia

The Innovative Care for Chronic Conditions (ICCC) Framework relies on a level of health workforce capacity that is often not available. In Malaysia, supporting professional staff like trained dieticians, exercise therapists, diabetic educators and other members of NCD team are not easily available, especially at the district level. The NCD team may consist of the doctor and nurses who may not be especially trained on NCD management. Many other middle income countries would face similar constraints.

Most of the health systems of middle-income countries, including Malaysia, are organised around models of healthcare developed in western countries which is care for episodic short term illness where patients can be isolated from the community and treated in healthcare centres. Such systems are clearly at odds when dealing with long-term and continuing illness that require collaboration across healthcare sectors and where patient behaviour change forms the primary focus.

Reorganizing health systems to ensure adequate care for the coming epidemic of chronic disease is clearly unaffordable especially if western models of care of chronic illness are to be adopted. This is especially so where practice teams may consists of a range of professionals like dieticians and diabetic educators.

In Malaysia, as previously discussed, the infrastructure exists to support chronic disease management at the community level. The community nurses who are currently trained to manage maternal and child health issues can, with adequate training, become effective practice teams for chronic disease care. The ability to access the population at the household level as well as the excellent rapport that they have with people at the community level are obvious advantages that can be used to implement the chronic care model.

### Extended range of services

To manage the high prevalence of NCDs, the government of Malaysia included an extended range of services in the health clinics or Klinik Kesihatan. These included treatment of both non-communicable (diabetes program, early detection of cancer) and communicable diseases (modified syndromic approach for sexually transmitted diseases, HIV screening, counselling and treatment). In addition, a wellness program was introduced (screening of women, screening of those above 40 years old for cardiovascular risk factors), as well as a tobacco cessation program, blindness prevention, mental health, elderly and adolescent health programs. KKs were free to introduce programs based on prevalence and health priorities in the area. Doctors and some nursing staff were sent for training prior to setting up these programs.

The extended services, however, did not include or extend to the community clinics (Klinik Desa) which were mainly manned by maternity nurses and who had access right down to the household levels [20].

The extended range of services introduced within the health system were not as successful in dealing with NCDs as compared with the earlier health system interventions that were introduced 30 years earlier for dealing with maternal and child health issues. NCDs prevalence has continued to rise.

There are a number of reasons for this. First, the major focus of the Malaysian primary health care system has been on maternal and child health and therefore the capacity did not exist at that level, for the management of chronic diseases. Re-orienting the system and staff was a major challenge especially without a significant increase in resources and personnel. Second, the public health system had a strong focus on communicable disease surveillance and control and on maternal and child health. There was a risk and worry that a major addition of duties may see a neglect of these conditions. Third, there was a lack of effective involvement of the community clinics (KDs) staff who had an intimate knowledge and access into the community. Finally, access issues may have been exacerbated by the growing private health sector. Chronic disease management often involves significant financial commitments and is likely to disadvantage those in lower income groups especially if they have to pay for healthcare [21]. This trend would also likely to increase access differences between rural and urban communities.

### Adapting effective strategies

In the face of such difficulties in re-aligning healthcare systems to manage NCDs, there may be value in adapting some strategies that have been shown to be effective in western settings and which may be more congruent with the social, cultural and healthcare environments of low-and-middle-income countries [22].

### Individual care of chronic disease: social support and self-management

Current models of chronic disease management focus on encouraging behaviour change at an individual level through improving self-management.

It is recognised that social support is a protective factor in health, for example for healthier aging [23], improved rehabilitation-related outcomes for heart patients [22] and better quality of life among chronic disease populations [24]. Social support can be defined into two broad categories: Structural (the number and type of social relationships) and functional social support (the perceived benefit of that support) [25]. Functional support can include perceived supportive relationships such as emotional support (like care, love and empathy) and social companionships but also includes instrumental, informational and appraisal support [26]. Lack of social support is associated with higher mortality and morbidity. This association has been shown in a range of varied conditions including cancer, depression and post myocardial infarction [27].

Self-management support has been defined as increasing skills and confidence of patients in managing their health problems through educational and supportive interventions. A WHO report has concluded that on a global scale, "Improving self-management of chronic diseases would have a far greater impact on health of the population than any improvement in specific medical treatments" [28].

Self-care or self-management strategies where patients take control for their care and treatment plans have fostered an atmosphere conducive to the use of peer support strategies and family-based interventions, both of which may be generalizable to low-and-middle-income countries.

### Peer support as one form of social support

A strategy that is being increasingly researched for improving social support has been to provide support through peers [29]. Peer support which has been defined by Dennis (2003) as "...provision of emotional, appraisal and informational assistance by a created social network member who possesses experiential knowledge of a specific behaviour or stressor and similar characteristics as a target population."[30] A peer, thus, shares common characteristics with the target group or individual, allowing him or her to relate to, and empathise with, that individual on a level that non-peers would not be able to. The main objective of peer support interventions is to provide functional support based on sharing of information and experience, mutual counselling and exchange among peers and this can contribute to sustained behaviour change.

The WHO consultation report on Peer Support Programmes in Diabetes acknowledges the value of peer support as an effective approach to chronic disease management and recommended further research [31]. Such research is especially important in low-and-middle-income countries. This is due to the applicability of such peer support programmes in less developed countries and the fact that it may be possible to implement these projects even in countries with poor health systems. One such program aimed at initiating and supporting further research around the world in peer support has been the Peer for Progress Program which is a global initiative of the American Academy of Family Physicians Foundation [29].

The evidence for peer support while not compelling is suggestive of benefit. A Cochrane review on self-management education programme by lay leaders for people with chronic conditions concluded that self-efficacy and self-rated health and cognitive symptom management as well as frequency of exercise improved in participants [32]. Another Cochrane review concluded that telephone peer support had some effect in changing patient behaviour over a range of conditions [33]. However, a recently published trial indicated that regular meetings with peer supporters at healthcare premises did not lead to improvement in outcomes [34].

It does appear that peer support is a complex intervention and may consist of many components. The exact component of the peer support process that may lead to improvement is yet unclear. The components may in fact be different in a developed country compared to a developing one and may be related to cultural and social factors.

### Family based interventions

Fisher (2000) noted "The life context that is the most pervasive, has the greatest, most long lasting effect on its members, and has the most influence on the management of type 2 diabetes is the family". Family relationships can influence outcomes in chronic disease by either directly affecting patient's physiological systems, like attachments and hostility or through family members' response support for to self-management [35,36]. One additional benefit of using family members is that behavioural change is likely to involve members other than the target member which has important implications for preventive care especially for chronic conditions where risk factors run in families. There is evidence from a systematic review by Armour et al. (2005) that interventions aimed at family members of people with diabetes is effective in improving glycaemic control [37].

In developing countries with traditional value systems, where the family relationships is paramount, and where government social support systems are often rudimentary using family members to provide support has special resonance.

## Conclusions

Chronic disease is a major cause of mortality and morbidity in developing countries and this is projected to increase. There are now well established, evidence-based strategies to control this pandemic. However, most of the evidence comes from studies conducted in well-resourced, well-functioning health systems and from countries with good social safety net. If we are to have any hope of dealing effectively with these conditions in countries like Malaysia, which has among the highest rates of NCDs in South East Asia, there is an urgent need to find innovative strategies to modify the current well established strategies to local conditions. These strategies need to be developed within the context of current debates on health care reforms and approaches to health systems strengthening; of financing models, of attention to universal coverage and equity and of the population at risk. Programs that encourage and support self-management including support from peers and from family members may be particularly useful.
